# Improving the management of chronic pain, opioid use, and opioid use disorder in older adults: study protocol for I-COPE study

**DOI:** 10.1186/s13063-022-06537-w

**Published:** 2022-07-27

**Authors:** Ainur Kagarmanova, Heather Sparkman, Neda Laiteerapong, Katherine Thompson, Linda Rosul, Danielle Lazar, Erin Staab, Wen Wan, Amanda Kass, Mim Ari

**Affiliations:** 1 Department of Medicine, University of Chicago, 5841 S. Maryland Ave., Rm. B200, MC 2007B, Chicago, IL 60637 USA; 2 Access Community Health Network, Chicago, IL USA

**Keywords:** Chronic pain, Opioid use, Older adults, Electronic health records, Primary health care

## Abstract

**Background:**

Older adults with chronic pain, opioid use, and opioid use disorder (OUD) present complex management decisions in primary care. Clinical tools are needed to improve care delivery. This study protocol describes the planned implementation and evaluation of I-COPE (Improving Chicago Older Adult Opioid and Pain Management through Patient-centered Clinical Decision Support and Project ECHO®) to improve care for this population.

**Methods:**

This study uses a pragmatic, expanding cohort stepped-wedge design to assess the outcomes. The study will be implemented in 35 clinical sites across metropolitan Chicago for patients aged ≥ 65 with chronic pain, opioid use, or OUD who receive primary care at one of the clinics. I-COPE includes the integration of patient-reported data on symptoms and preferences, clinical decision support tools, and a shared decision-making tool into routine primary care for more effective management of chronic pain, opioid prescribing, and OUD in older adults. Primary care providers will be trained through web-based videos and an optional Project ECHO® course, entitled “Pain Management and OUD in Older Adults.” The RE-AIM framework will be used to assess the I-COPE implementation. Effectiveness outcomes will include an increased variety of recommended pain treatments, decreased prescriptions of higher-risk pain treatments, and decreased patient pain scores. All outcomes will be evaluated 6 and 12 months after implementation. PCPs participating in Project ECHO® will be evaluated on changes in knowledge, attitudes, and self-efficacy using pre- and post-course surveys.

**Discussion:**

This study will provide evidence about the effectiveness of collecting patient-reported data on symptoms and treatment preferences and providing clinical decision support and shared decision-making tools to improve management for older adults with chronic pain, opioid use, and OUD.

**Trial registration:**

ClinicalTrials.gov NCT04878562.

**Supplementary Information:**

The online version contains supplementary material available at 10.1186/s13063-022-06537-w.

## Administrative information

Sections of this protocol are not numbered with SPIRIT Checklist item numbers, please refer to Additional file 7 for the completed SPIRIT Checklist. Participant timeline is presented in Table 1.

**Table 1:** Schedule of enrollment, interventions, and assessments for the I-COPE Project

	Study period								
	Enrollment	Implementation							Close-out
Time point	*March 21*	*September 21*	*November 21*	*January 22*	*March 22*	*July 22*	*September 22*	*November 22*	*November 23*
Enrollment									
Patients			x	x	x	x	x	x	x
Providers	x	x	x	x	x	x			
Interventions									
* Project ECHO Course: I-COPE Step 1*		x	x						
* Project ECHO Course: I-COPE Step 2*				x					
* Project ECHO Course: I-COPE Step 3*				x	x				
* Project ECHO Course: I-COPE Step 4*					x				
* Project ECHO Course: I-COPE Step 5*							x		
* I-COPE Toolkit: implementation step 1*			x	x	x	x	x	x	x
* I-COPE Toolkit: implementation step 2*				x	x	x	x	x	x
* I-COPE Toolkit: implementation step 3*					x	x	x	x	x
* I-COPE Toolkit: implementation step 4*						x	x	x	x
* I-COPE Toolkit: implementation step 5*								x	x
Assessments	x								
* I-COPE toolkit usage*		x	x	x	x	x	x	x	x
* Diversity of provider recommended pain treatments*		x	x	x	x	x	x	x	x
* Prescribed higher-risk pain treatments*		x	x	x	x	x	x	x	x
* Patient-reported pain scores*		x	x	x	x	x	x	x	x
* Provider self-efficacy*		x	x	x	x	x			

The World Health Organization Trial Registration Dataset.

**Table:** 

Data category	Information^32^
Primary registry and trial identifying number	ClinicalTrials.gov NCT04878562
Date of registration in primary registry	7 May 2021
Secondary identifying numbers	IRB20-1580
Source(s) of monetary or material support	Agency for Healthcare Research and Quality
Primary sponsor	Agency for Healthcare Research and Quality
Secondary sponsor(s)	n/a
Contact for public queries	Ainur Kagarmanova, MS [akagarmanova@medicine.bsd.uchicago.edu] University of Chicago
Contact for scientific queries	Mim Ari, MD [mari2@medicine.bsd.uchicago.edu] University of Chicago
Public title	Improving the management of chronic pain, opioid use, and opioid use disorder in older adults (I-COPE): study protocol
Scientific title	Improving the management of chronic pain, opioid use, and opioid use disorder in older adults (I-COPE): study protocol
Countries of recruitment	United States
Health condition(s) or problem(s) studied	Chronic pain, opioid use, opioid use disorder
Intervention(s)	Active comparator: *ICOPE intervention*
	Placebo comparator: n/a
Key inclusion and exclusion criteria	Ages eligible for the study: ≥ 65 years Sexes eligible for the study: both Accepts healthy volunteers: no
	Inclusion criteria: older adult patient (≥ 65 years), diagnosed with chronic pain or conditions associated with chronic pain, high pain Score (> 7) at a previous visit opioid use disorder and/or current opioid use
	Exclusion criteria: none
Study type	Interventional
	Allocation: randomized
	Primary purpose: health services research
	Phase: n/a
Date of first enrolment	June 2021
Target sample size	3040
Recruitment status	Recruiting
Primary outcome(s)	Variety of recommended pain treatments; prescribed higher-risk treatments; patient pain scores
Key secondary outcomes	Reach and adoption of I-COPE tools; safe opioid prescribing measures, primary care providers’ knowledge, attitudes pre- and post- ECHO Chicago course, self-efficacy related to managing older adults with chronic pain, opioid use, and OUD

## Background

Adults aged 65 years or older experience more pain than younger adults, with approximately half of older adults in the USA experiencing chronic pain [1–3]. Chronic pain in older adults can be complex and challenging for primary care providers (PCPs) to manage because older adults are at higher risk for poor outcomes related to multimorbidity and polypharmacy. To manage chronic pain in older adults, opioids may be appropriate. However, caution is needed due to older adults having a higher risk for side effects, greater frequency of opioid-related emergency department visits, and increases in both heroin use and opioid overdose deaths [4, 5].

To facilitate the management of chronic pain in older adults, several key factors need to be incorporated [6, 7]. From the patient standpoint, a thorough understanding of each patient’s goals and preferences, comorbidities, and resources is necessary. Additionally, coordination of care team members and caregivers is important, and usually, combinations of different types of therapies are needed [8]. However, implementation of clinical guidelines in practice can be difficult. Limited visit time, patient and PCP reluctance to change an established routine, and lack of PCP education and training prevent optimal care for chronic pain in older adults [9]. To help improve chronic pain management for older adults, better tools are needed [1, 2, 10, 11]. I-COPE was designed to address these issues through the collection of patient-reported data on symptoms and preferences, and implementation of clinical decision support and shared decision-making tools, with accompanying PCP education.

## Methods

### Intervention study aims

Improving Chicago Older Adult Opioid and Pain Management through PCCDS and Project ECHO® (I-COPE) aims to leverage patient-reported data on symptoms and preferences, clinical decision support tools, and a shared decision-making tool to improve clinical management of chronic pain, opioid prescribing, and OUD in older adults.

### Study design

**Fig. 1:**
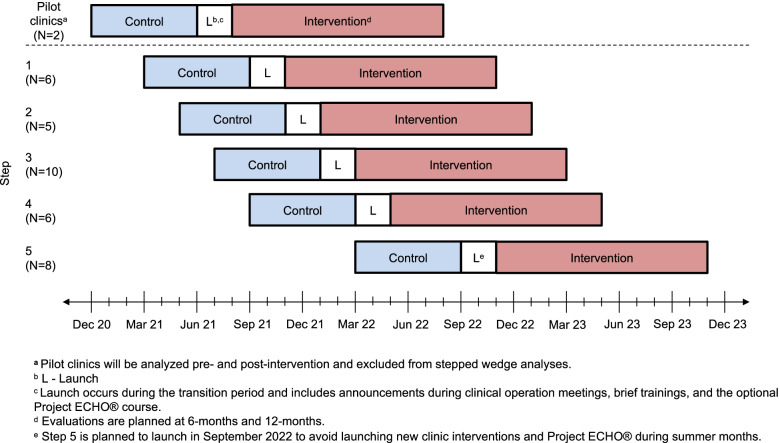
I-COPE study design: stepped-wedge study with a transition period

As part of the development phase of I-COPE, the full workflow and EHR tools are being piloted at two University of Chicago Medicine (UCM) clinics. Pilot phase implementation experiences are being used to improve the I-COPE tools and processes. I-COPE will be implemented in a pragmatic, expanding cohort stepped-wedge trial with a transition period, continuous recruitment, and longitudinal exposure (Fig. 1) [12]. There will be a total of five, 12-week steps with 5 to 9 clinics per step, starting September 2021. All five steps are expected to implement I-COPE by November 2022.

### Study setting and participants

The study will be implemented at 35 clinical sites in the Chicago metropolitan area from Access Community Health Network (ACCESS) or UCM. ACCESS is a federally qualified health center (FQHC) network with 35 clinical sites; all clinical sites that care for older adults (*N* = 32) are participating in this study. UCM is an academic medical center with affiliated community clinics, and 3 out 4 primary care clinics agreed to participate. PCPs include physicians, physician assistants, and advanced practice nurses. Epic® electronic health record (EHR)-based tools for I-COPE were built for both health systems and are available for all PCPs at participating sites. Eligible patients will be identified based on pre-existing EHR data. The inclusion criteria are patients 65 years or older who receive primary care at a study site during the study period and have a pain score ≥ 6 or have diagnoses frequently associated with chronic pain (full ICD-10 list provided in Additional file 1), a current opioid prescription, or have been diagnosed with OUD (full ICD-10 list provided in Additional file 2) (Table 2).

**Table 2:** I-COPE study patient inclusion criteria

Criteria^a^	Definition
Age	≥ 65 years
Visit type	Virtual or in-person primary care visits
Chronic pain	• Last visit pain score ≥ 6 in the last 12 months • Diagnosis associated with chronic pain on problem list or past encounter in the last 12 months
Opioid use	Two or more opioid prescriptions in the last 12 months
OUD [2]	OUD diagnosis on problem list, past medical history, or any past encounter

*Abbreviation: OUD*, opioid use disorder

^a^Patients eligible for inclusion must meet age and visit type criteria and at least one of the criteria for chronic pain, opioid use, or OUD definitions

### Intervention

#### Development of I-COPE

I-COPE was developed using an iterative design approach over a 9-month period. The design of I-COPE was led by a multidisciplinary team of investigators with input from a stakeholder advisory panel. The I-COPE team includes experts in geriatrics, palliative medicine, primary care, clinical informatics, shared decision-making, medical education, addiction medicine, behavioral medicine, implementation science, and clinical research. The stakeholder advisory panel included additional content experts, as well as three older adult patients. In addition, specialists in the Epic® EHR system were involved.

#### I-COPE tools

I-COPE includes a pre-visit patient questionnaire that PCPs can review to enable shared decision making, alongside clinical decision support, to create a comprehensive pain, opioid, and opioid use disorder management plan.

##### Patient-reported data on symptoms and preferences (pre-visit questionnaire)

*Questionnaire contents.* Eligible patients receive a pre-visit questionnaire that asks about pain symptoms, goals for pain management, pain treatment preferences, depression symptoms, and opioid and illicit drug use (Additional file 3). The questionnaire uses validated screeners to ask about current levels of pain (Pain, Enjoyment of Life, General Activity (PEG) scale), depression (PHQ-2), and drug use (drug abuse screening test (DAST-2) (ACCESS) or single-question drug use screener (UCM)) [13–15]. The I-COPE questionnaire also asks patients to identify functional goals that might be achieved through better pain management, and treatment preferences ranging from self-management to surgical intervention.

*Questionnaire administration.* The pre-visit questionnaire will be automatically assigned to eligible patients when they schedule a clinic appointment. Three days before their clinic appointment, eligible patients who have an active patient portal account will be invited to complete the pre-visit questionnaire. If patients do not complete the questionnaire before their appointment or they do not have an active patient portal account, they will be asked to complete the questionnaire at check-in either on paper or using electronic tablets, depending on the workflow in that clinic. Medical assistants will enter paper responses into the Epic® EHR. A caregiver may help the patient complete the questionnaire if necessary.

##### Clinical decision support tool (adaptive order set)

Completion of a pre-visit questionnaire by patients will automatically generate a passive alert (best practice advisory) to PCPs. The alert will summarize the questionnaire responses and prompt the PCP to open an order set (Additional file 4). An electronic order set is a tool designed to streamline ordering and documentation in the clinical setting, with the goal of presenting the right information to the right person at the right time in the right point in the workflow [16]. The I-COPE order set was designed based on the American Geriatrics Society “Geriatrics At Your Fingertips” pain management chapter and the CDC guideline for prescribing opioids for chronic pain [6, 7]. The order set includes seven sections: self-management (patient education handouts on non-pharmacologic pain management strategies, exercises, assistive devices), referrals to other specialists, topical medications, oral non-opioid medications, medications for acute-on-chronic pain, opioid medications, and OUD. Each of these sections includes brief guidance and links to additional resources. Associated orders are customized and pre-filled. Three out of seven sections of the order set are designed to adapt to pre-visit patient questionnaire responses and data from the EHR. For instance, if a patient screens positive for depression or has a history of depression, the *antidepressants* group within the *oral non-opioid medications* section will open, nudging towards this medication class. Alternative versions of other sections exist for patients with advanced chronic kidney disease, opioid or benzodiazepine prescriptions, positive drug use screening results, and a previous diagnosis of OUD.

##### Shared decision-making tool (conversation tool)

The I-COPE conversation tool is a one-page document that lists evidence-based examples of pain management strategies for older adults (Additional file 5). The conversation tool supports shared decision-making by offering a visually compelling summary of available approaches in simple language [17]. It is intended to facilitate the conversation about treatment options between the patient and PCP at the time of the visit and mirrors the options available in the electronic order set.

#### I-COPE training/education

Prior to the launch of I-COPE at participating clinics, the new clinical workflows and tools are announced at a clinical operations meeting during which PCPs are informed about training opportunities and encouraged to participate. Electronic and printed tip sheets are provided for PCPs and staff members. Training options include (1) self-directed brief online videos walking through the I-COPE tools and general principles of older adult pain management and shared decision-making or 2) an 8-session Project ECHO® (Extension for Community Health Outcomes) series entitled Pain Management and OUD in Older Adults. The local version of Project ECHO® at the University of Chicago is ECHO-Chicago. Each session is an hour long and includes a 30-min didactic topic delivered by a content expert and two 15-min discussions of real-world cases brought by participating PCPs. Project ECHO® is an innovative workforce development model for expanding primary care capacity in underserved communities [18–21]. It uses videoconferencing technology to “telementor” community-based clinicians via didactic education, group problem-solving with actual cases brought by clinicians, and expert advice on implementing best practices [22]. These clinicians then become a “force multiplier” as they serve as local experts. Participating PCPs receive continuing medical education credits.

### Randomization

Due to potential study arm contamination from certain PCPs who work at multiple sites, the 32 ACCESS sites and 3 UCM sites were bundled into 22 units (19 ACCESS and three UCM). We then randomized the 22 units into five steps, stratified by affiliation (ACCESS vs UCM) to balance the number of PCPs from ACCESS and UCM sites within each step. We also minimized sequential imbalance across multiple site-level characteristics by including volume and percentages of racial groups such as White, Black, Hispanic, Asian, and others [23]. We chose the randomization with the smallest total imbalance score of the characteristics, in which each characteristic was rescaled into a standard normal distribution to be on the same scale. Clusters were numbered one through five and the allocation sequence was concealed until clusters were assigned. The I-COPE study biostatistician (co-author Wen Wan) generated the allocation sequence.

### Implementation plan

For each step, preparation of participating clinical sites for I-COPE implementation includes general information sessions on I-COPE, online training materials for involved PCPs and staff, and in-clinic tip sheets. PCPs are recruited for voluntary participation in Project ECHO® ahead of implementation. Once launched, the I-COPE study staff provide support and identify workflow issues. Completion of assigned pre-visit questionnaires and acknowledgement of completed questionnaires are tracked to monitor implementation progress.

### Intervention outcomes

The RE-AIM framework will be used to assess the effectiveness of the implementation of I-COPE (Table 3) [24]. Effectiveness outcomes will include an increase in the variety of recommended pain treatments at the clinic and PCP level, decrease in prescribed higher-risk pain treatments (discontinuation or decrease in prescribed daily milligram equivalents of opioids and Beer’s criteria medications), and decreases in patient pain scores (% of patients with high initial pain scores (≥ 6) who experience a 30% reduction in scores) at 6 months (Table 3). Additional outcomes include measures of use (reach and adoption) of I-COPE tools by PCPs and clinics, specific safe opioid prescribing measures, and evaluation of Project ECHO® participants through pre- and post-surveys that include change in PCP knowledge, attitudes, and self-efficacy related to managing older adults with chronic pain, opioid use, and OUD (Table 3). Outcomes will be evaluated again at 12 months to assess sustainability.

**Table 3:** RE-AIM framework implementation outcomes used to evaluate the ICOPE program implementation

Framework dimension	Outcome
Reach	• # and % of eligible patients who complete the pre-visit questionnaire • # and % of eligible PCPs who use the I-COPE order set • # and % of PCPs participating in ECHO-Chicago
Effectiveness	• Change in a variety of recommended pain treatments • Change in prescribing of higher-risk pain treatments (opioids and Beer’s criteria medications) • Change in pain scores o % with chronic pain diagnoses and high initial pain scores (≥ 6) who experience a 30% reduction in scores in 6 months • Change in safe opioid prescribing practices o Annual drug screens in a patient with chronic opioid use o Naloxone prescribing in patients with > 50 MME equivalents of opioids or OUD o Co-prescribing of opioids and benzodiazepines • Change in self-efficacy and practice behaviors for ECHO-Chicago participants
Adoption	• # and % of clinics who use the I-COPE Program
Implementation	• # and % of eligible patients who received all I-COPE tools (pre-visit questionnaire and order set use) • # and % of PCPs who participated in ECHO-Chicago and attended all eight sessions
Maintenance	• Outcomes listed above at 12 months

*Abbreviation: PCP* primary care providers, *ECHO-Chicago* Extension for Community Health Outcomes-Chicago, *MME* morphine milligram equivalents, *OUD* opioid use disorder, *I-COPE* Improving Chicago Older Adult Opioid and Pain Management Through Patient-centered Clinical Decision Support and Project ECHO®

### Data collection

#### Clinics

We will collect descriptive characteristics about clinical sites, including size (i.e., number of patients and visits per year); number of PCPs and staff; PCP characteristics, including sex, race, ethnicity, and years in practice; and clinical resources (e.g., integrated behavioral health). Data extracted from Epic® will be used to calculate the use of the I-COPE tools by PCPs.

#### Patients

We will obtain data on all eligible patients who receive care at study sites during the study period, which is defined as 6 months prior to launch at the step 1 clinical sites through 12 months after the step 5 launch. We will collect de-identified patient pre-visit questionnaire responses, data on patient characteristics (e.g., age, gender, race, ethnicity, insurance), diagnoses, pain scores, medications, referrals, other treatments, mental health assessments, and indicators of safe opioid prescribing (e.g., urine drug screens, naloxone scripts).

### Sample size

We calculated the sample size for the effectiveness outcome that had pre-intervention data, change in patient pain scores (% of patients with high initial pain scores (≥ 6) who experience a 30% reduction in scores) at 6 months, using the sample size calculation method for closed cohort (a special case of expanding cohort) by Hooper et al. [25] and Hooper and Bourke [26]. We estimated that there were an average of 26 PCPs and about 600 patients per step. We expect that at least 68% of older adults with pain scores ≥ 6 experiencing ≥ 30% pain score reduction due to the intervention. The total sample of 3040 eligible patients within 5 steps is needed to ensure at least 80% power to detect the difference of 10% at a one-sided significance level of 5%. Given a total of > 3400 patients with baseline pain score ≥ 6 in the 35 sites, the power becomes much larger. More detailed information is available in the Additional file 6.

### Data analysis

An intention-to-treat (ITT) analysis principle will be applied to all outcomes. For analyses of the effectiveness outcomes, we will use the blended exchangeable correlation structure [27] via generalized estimating equation (GEE) and generalized linear mixed-effects model (GLMM) to evaluate the intervention effect over 2 years since the beginning of the program implementation. For analyses of outcomes that require data collection provided by the intervention, such as reach, adoption, and implementation outcomes, we will provide basic descriptive statistics per clinical site per period and over all clinical sites and all periods. The study results will be released to the participating physicians, referring physicians, patients, and the general medical community.

## Discussion

### Expected impact/significance

Older adults disproportionately experience chronic pain and are vulnerable to adverse consequences of both undertreated pain and from pain treatments, including opioid use. However, limited evidence exists on how to implement optimal pain management for older adults in a primary care setting. This novel study will be one of the first to integrate patient-reported data on symptoms and preferences, a clinical decision support tool, and a shared decision-making tool for older adults with chronic pain, opioid use, and OUD. We also use a pragmatic design to test real-world implementation and effectiveness. The stepped-wedge design allows for a staged rollout across 35 clinical sites within two health systems and assessment of the effectiveness of the program in existing clinical practice while controlling for secular trends. I-COPE uses ECHO-Chicago to distribute knowledge to PCPs about the I-COPE components and geriatrics management principles for older adults with chronic pain, opioid use, and OUD. Our work suggests that ECHO-Chicago improves self-efficacy and practice behaviors in geriatrics competencies for urban medical PCPs [28–30]. While I-COPE targets older adults, given the ongoing issue of how to address chronic pain, opioid use, and OUD more broadly, we believe that the results of this study and the resulting tools will have potential application in other age ranges and populations.

### Limitations

Findings may not generalize to non-urban populations or in settings with less availability to resources such as those offered by ACCESS and UCM clinical sites (access to behavioral health, PCP access to education, etc.).

## Conclusions

The goal of this study is to determine whether the implementation of I-COPE will improve clinical care for Chicago’s older adults with chronic pain, opioid use, and OUD. The integration of patient-reported data on symptoms and preferences, clinical decision support tools and a shared decision-making tool is innovative. Our results will be generalizable to other clinics that care for urban older adults. Lessons learned from this study could add to the evidence for the necessary components to effectively improve management for older adults with chronic pain, opioid use, or opioid use disorder.

## Supplementary Information

**Additional file 1. **ICD-10 codes for chronic pain and related conditions.

**Additional file 2.** ICD-10 codes for opioid use disorder and related conditions.

**Additional file 3.** Improving Chicago Older Adult Opioid and Pain Management Through Patient-centered Clinical Decision Support and Project ECHO (I-COPE) Patient Pre-Visit Questionnaire.

**Additional file 4.** Improving Chicago Older Adult Opioid and Pain Management Through Patient-centered Clinical Decision Support and Project ECHO (I-COPE) Electronic order set.

**Additional file 5.** Improving Chicago Older Adult Opioid and Pain Management Through Patient-centered Clinical Decision Support and Project ECHO (I-COPE) Conversation tool.

**Additional file 6.** Notes on sample size calculation and data analysis.

**Additional file 7.** SPIRIT Checklist.

## Data Availability

The datasets used and/or analyzed during the current study will be made available from the corresponding author upon reasonable request.
