# Supplementary material for: Improving the management of chronic pain, opioid use, and opioid use disorder in older adults: study protocol for I-COPE study
Source: Trials. 2022 Jul 27;23:602. doi: 10.1186/s13063-022-06537-w (PMC9327217; doi:10.1186/s13063-022-06537-w)
Supplement: Supplementary file 1 — Additional file 1. ICD-10 codes for chronic pain and related conditions. [file 13063_2022_6537_MOESM1_ESM.docx]

**Additional file 1**

ICD-10 codes for chronic pain related conditions

| **ICD-10-CM** | **Name** |
| --- | --- |
| B02.2 | Zoster with other nervous system involvement |
| B02.21 | Postherpetic geniculate ganglionitis |
| B02.22 | Postherpetic trigeminal neuralgia |
| B02.23 | Postherpetic polyneuropathy |
| B02.24 | Postherpetic myelitis |
| B02.29 | Other postherpetic nervous system involvement |
| E08.4 | Diabetes mellitus due to underlying condition with neurological complications |
| G43.019 | Migraine without aura, with intractable migraine, so stated, without mention of status migrainosus |
| G43.101 | Migraine with aura, without mention of intractable migraine with status migrainosus |
| G43.109 | Migraine with aura, without mention of intractable migraine without mention of status migrainosus |
| G43.109, R56.9 | Migraine triggered seizures |
| G43.119 | Migraine with aura, with intractable migraine, so stated, without mention of status migrainosus |
| G43.409 | Hemiplegic migraine |
| G43.50 | Persistent migraine aura without cerebral infarction, not intractable |
| G43.51 | Persistent migraine aura without cerebral infarction, intractable |
| G43.709 | Chronic migraine without aura, without mention of intractable migraine without mention of status migrainosus |
| G43.801 | Variants of migraine, not elsewhere classified, without mention of intractable migraine with status migrainosus |
| G43.809 | Variants of migraine, not elsewhere classified, without mention of intractable migraine without mention of status migrainosus |
| G43.811 | Variants of migraine, not elsewhere classified, with intractable migraine, so stated, with status migrainosus |
| G43.819 | Variants of migraine, not elsewhere classified, with intractable migraine, so stated, without mention of status migrainosus |
| G43.829 | Menstrual migraine |
| G43.901 | Status migrainosus |
| G43.909 | Migraine, unspecified, without mention of intractable migraine without mention of status migrainosus |
| G43.919 | Migraine, unspecified, with intractable migraine, so stated, without mention of status migrainosus |
| G43.B0 | Ophthalmoplegic migraine |
| G43.B1 | Ophthalmoplegic migraine, intractable |
| G43.C0 | Periodic headache syndrome |
| G43.C1 | Intractable periodic headache syndrome |
| G43.D0 | Abdominal migraine |
| G43.D1 | Abdominal migraine, intractable |
| G44.009 | Cluster headache |
| G44.201 | Intractable tension-type headache |
| G44.209 | Tension headache |
| G44.211 | Intractable episodic tension-type headache |
| G44.219 | Episodic tension type headache |
| G44.22 | Chronic tension-type headache |
| G50.0 | Trigeminal neuralgia |
| G50.1 | Atypical facial pain |
| G54.6 | Phantom limb syndrome with pain |
| G89.2 | Chronic pain, not elsewhere classified |
| G89.3 | Neoplasm related pain (acute) (chronic) |
| G89.4 | Chronic pain syndrome |
| G90.5 | Complex regional pain syndrome I (CRPS I) |
| I63.9, G43.909 | Stroke with migraine |
| I67.2, L65.9, M54.5 | Familial arteriosclerotic leukoencephalopathy with alopecia and lumbago, without arterial hypertension |
| K58.0 | Irritable bowel syndrome with diarrhea |
| K58.1 | Irritable bowel syndrome with constipation |
| K58.2 | Irritable bowel syndrome with constipation and diarrhea |
| K58.8 | Other irritable bowel syndrome |
| K58.9 | Irritable bowel syndrome |
| L40.5 | Arthropathic psoriasis |
| M05 | Rheumatoid arthritis with rheumatoid factor |
| M05.0 | Felty's syndrome |
| M05.1 | Rheumatoid lung disease with rheumatoid arthritis |
| M05.2 | Rheumatoid vasculitis with rheumatoid arthritis |
| M05.3 | Rheumatoid heart disease with rheumatoid arthritis |
| M05.4 | Rheumatoid myopathy with rheumatoid arthritis |
| M05.5 | Rheumatoid polyneuropathy with rheumatoid arthritis |
| M05.6 | Rheumatoid arthritis with involvement of other organs and systems |
| M05.7 | Rheumatoid arthritis with rheumatoid factor without organ or systems involvement |
| M05.8 | Other rheumatoid arthritis with rheumatoid factor |
| M05.9 | Rheumatoid arthritis with rheumatoid factor, unspecified |
| M15 | Polyosteoarthritis |
| M16 | Osteoarthritis of hip |
| M17 | Osteoarthritis of knee |
| M18 | Osteoarthritis of first carpometacarpal joint |
| M19 | Other and unspecified osteoarthritis |
| M26.60 | Temporomandibular joint disorder, unspecified |
| M26.62 | Arthralgia of temporomandibular joint |
| M26.63 | Articular disc disorder of temporomandibular joint |
| M45 | Ankylosing spondylitis |
| M54.1 | Radiculopathy |
| M54.2 | Cervicalgia |
| M54.3 | Sciatica |
| M54.4 | Lumbago with sciatica |
| M54.5 | Lumbago |
| M54.5, G89.29 | Chronic bilateral low back pain, unspecified whether sciatica present |
| M54.6 | Pain in thoracic spine |
| M54.6, M54.5 | Dorsalgia of thoracolumbar region |
| M54.8 | Other dorsalgia |
| M54.9 | Dorsalgia, unspecified |
| M79.0, M79.7 | Rheumatism, unspecified and fibrositis |
| M79.7 | Fibromyalgia |
| N30.10 | Chronic interstitial cystitis |
| N30.30 | Trigonitis |
| N41.1 | Chronic prostatitis |
| N94.810 | Vulvar vestibulitis |
| R53.82 | Chronic fatigue syndrome |
| R53.82, D89.89 | Chronic fatigue and immune dysfunction syndrome |
| R53.82, M79.7 | Chronic fatigue fibromyalgia syndrome |
| R53.82, M79.7 | Chronic fatigue syndrome with fibromyalgia |
| S34 | Injury of lumbar and sacral spinal cord and nerves at abdomen, lower back and pelvis level |
| S34.0 | Concussion and edema of lumbar and sacral spinal cord |
| S34.1 | Other and unspecified injury of lumbar and sacral spinal cord |
| S34.2 | Injury of nerve root of lumbar and sacral spine |
| S34.3 | Injury of cauda equina |
| S34.4 | Injury of lumbosacral plexus |
| S34.5 | Injury of lumbar, sacral and pelvic sympathetic nerves |
| S34.6 | Injury of peripheral nerve(s) at abdomen, lower back and pelvis level |
| S34.8 | Injury of other nerves at abdomen, lower back and pelvis level |
| S34.9 | Injury of unspecified nerves at abdomen, lower back and pelvis level |
