# Supplementary material for: Improving the management of chronic pain, opioid use, and opioid use disorder in older adults: study protocol for I-COPE study
Source: Trials. 2022 Jul 27;23:602. doi: 10.1186/s13063-022-06537-w (PMC9327217; doi:10.1186/s13063-022-06537-w)
Supplement: Supplementary file 2 — Additional file 2. ICD-10 codes for opioid use disorder and related conditions. [file 13063_2022_6537_MOESM2_ESM.docx]

**Additional file 2**

ICD-10 codes for opioid use disorder and related conditions

| ICD-10 codes | Name |
| --- | --- |
| F11.10 | Opioid abuse, uncomplicated |
| F11.120 | Opioid abuse with intoxication, uncomplicated |
| F11.121 | Opioid abuse with intoxication, delirium |
| F11.122 | Opioid abuse with intoxication, with perceptual disturbance |
| F11.129 | Opioid abuse with intoxication, unspecified |
| F11.14 | Opioid abuse with opioid-induced mood disorder |
| F11.150 | Opioid abuse with opioid-induced psychotic disorder, with delusions |
| F11.151 | Opioid abuse with opioid-induced psychotic disorder, with hallucinations |
| F11.159 | Opioid abuse with opioid-induced psychotic disorder, unspecified |
| F11.181 | Opioid abuse with opioid-induced sexual dysfunction |
| F11.182 | Opioid abuse with opioid-induced sleep disorder |
| F11.188 | Opioid abuse with other opioid-induced disorder |
| F11.19 | Opioid abuse with unspecified opioid-induced disorder |
| F11.20 | Opioid dependence, uncomplicated |
| F11.21 | Opioid dependence, in remission |
| F11.220 | Opioid dependence with intoxication, uncomplicated |
| F11.221 | Opioid dependence with intoxication, delirium |
| F11.222 | Opioid dependence with intoxication, with perceptual disturbance |
| F11.229 | Opioid dependence with intoxication, unspecified |
| F11.23 | Opioid dependence with withdrawal |
| F11.24 | Opioid dependence with opioid-induced mood disorder |
| F11.250 | Opioid dependence with opioid-induced psychotic disorder, with delusions |
| F11.251 | Opioid dependence with opioid-induced psychotic disorder, with hallucinations |
| F11.259 | Opioid dependence with opioid-induced psychotic disorder, unspecified |
| F11.281 | Opioid dependence with opioid-induced sexual dysfunction |
| F11.282 | Opioid dependence with opioid-induced sleep disorder |
| F11.288 | Opioid dependence with other opioid-induced disorder |
| F11.29 | Opioid dependence with unspecified opioid-induced disorder |
| **Opioid use** | |
| F11.90 | Opioid use, unspecified, uncomplicated |
| F11.920 | Opioid use, unspecified with intoxication, uncomplicated |
| F11.921 | Opioid use, unspecified with intoxication delirium |
| F11.922 | Opioid use, unspecified with intoxication, with perceptual disturbance |
| F11.929 | Opioid use, unspecified with intoxication, unspecified |
| F11.93 | Opioid use, unspecified, with withdrawal |
| F11.94 | Opioid use, unspecified, with opioid-induced mood disorder |
| F11.950 | Opioid use, unspecified with opioid-induced psychotic disorder, with delusions |
| F11.951 | Opioid use, unspecified with opioid-induced psychotic disorder, with hallucinations |
| F11.959 | Opioid use, unspecified with opioid-induced psychotic disorder, unspecified |
| F11.981 | Opioid use, unspecified with opioid-induced sexual dysfunction |
| F11.982 | Opioid use, unspecified with opioid-induced sleep disorder |
| F11.988 | Opioid use, unspecified with other opioid-induced disorder |
| F11.99 | Opioid use, unspecified, with unspecified opioid-induced disorder |
| **Poisoning** | |
| T40.0X1A | Poisoning by opium, accidental (unintentional), initial encounter |
| T40.0X1D | Poisoning by opium, accidental (unintentional), subsequent encounter |
| T40.0X2A | Poisoning by opium, intentional self-harm, initial encounter |
| T40.0X2D | Poisoning by opium, intentional self-harm, subsequent encounter |
| T40.0X3A | Poisoning by opium, assault, initial encounter |
| T40.0X3D | Poisoning by opium, assault, subsequent encounter |
| T40.0X4A | Poisoning by opium, undetermined, initial encounter |
| T40.0X4D | Poisoning by opium, undetermined, subsequent encounter |
| T40.1X1A | Poisoning by heroin, accidental (unintentional), initial encounter |
| T40.1X1D | Poisoning by heroin, accidental (unintentional), subsequent encounter |
| T40.1X2A | Poisoning by heroin, intentional self-harm, initial encounter |
| T40.1X2D | Poisoning by heroin, intentional self-harm, subsequent encounter |
| T40.1X3A | Poisoning by heroin, assault, initial encounter |
| T40.1X3D | Poisoning by heroin, assault, subsequent encounter |
| T40.1X4A | Poisoning by heroin, undetermined, initial encounter |
| T40.1X4D | Poisoning by heroin, undetermined, subsequent encounter |
| T40.2X1A | Poisoning by other opioids, accidental (unintentional), initial encounter |
| T40.2X1D | Poisoning by other opioids, accidental (unintentional), subsequent encounter |
| T40.2X2A | Poisoning by other opioids, intentional self-harm, initial encounter |
| T40.2X2D | Poisoning by other opioids, intentional self-harm, subsequent encounter |
| T40.2X3A | Poisoning by other opioids, assault, initial encounter |
| T40.2X3D | Poisoning by other opioids, assault, subsequent encounter |
| T40.2X4A | Poisoning by other opioids, undetermined, initial encounter |
| T40.2X4D | Poisoning by other opioids, undetermined, subsequent encounter |
| T40.3X1A | Poisoning by methadone, accidental (unintentional), initial encounter |
| T40.3X1D | Poisoning by methadone, accidental (unintentional), subsequent encounter |
| T40.3X2A | Poisoning by methadone, intentional self-harm, initial encounter |
| T40.3X2D | Poisoning by methadone, intentional self-harm, subsequent encounter |
| T40.3X3A | Poisoning by methadone, assault, initial encounter |
| T40.3X3D | Poisoning by methadone, assault, subsequent encounter |
| T40.3X4A | Poisoning by methadone, undetermined, initial encounter |
| T40.3X4D | Poisoning by methadone, undetermined, subsequent encounter |
| T40.4X1A | Poisoning by synthetic narcotics, accidental (unintentional), initial encounter |
| T40.4X1D | Poisoning by synthetic narcotics, accidental (unintentional), subsequent encounter |
| T40.4X2A | Poisoning by other synthetic narcotics, intentional self-harm, initial encounter |
| T40.4X2D | Poisoning by other synthetic narcotics, intentional self-harm, subsequent encounter |
| T40.4X3A | Poisoning by other synthetic narcotics, assault, initial encounter |
| T40.4X3D | Poisoning by other synthetic narcotics, assault, subsequent encounter |
| T40.4X4A | Poisoning by synthetic narcotics, undetermined, initial encounter |
| T40.4X4D | Poisoning by synthetic narcotics, undetermined, subsequent encounter |
| T40.601A | Poisoning by unspecified narcotics, accidental (unintentional), initial encounter |
| T40.601D | Poisoning by unspecified narcotics, accidental (unintentional), subsequent encounter |
| T40.602A | Poisoning by unspecified narcotics, intentional self-harm, initial encounter |
| T40.602D | Poisoning by unspecified narcotics, intentional self-harm, subsequent encounter |
| T40.603A | Poisoning by unspecified narcotics, assault, initial encounter |
| T40.603D | Poisoning by unspecified narcotics, assault, subsequent encounter |
| T40.604A | Poisoning by unspecified narcotics, undetermined, initial encounter |
| T40.604D | Poisoning by unspecified narcotics, undetermined, subsequent encounter |
| T40.691A | Poisoning by other narcotics, accidental (unintentional), initial encounter |
| T40.691D | Poisoning by other narcotics, accidental (unintentional), subsequent encounter |
| T40.692A | Poisoning by other narcotics, intentional self-harm, initial encounter |
| T40.692D | Poisoning by other narcotics, intentional self-harm, subsequent encounter |
| T40.693A | Poisoning by other narcotics, assault, initial encounter |
| T40.693D | Poisoning by other narcotics, assault, subsequent encounter |
| T40.694A | Poisoning by other narcotics, undetermined, initial encounter |
| T40.694D | Poisoning by other narcotics, undetermined, subsequent encounter |
| **Adverse effects** | |
| T40.0X5A | Adverse effect of opium, initial encounter |
| T40.0X5D | Adverse effect of opium, subsequent encounter |
| T40.2X5A | Adverse effect of other opioids, initial encounter |
| T40.2X5D | Adverse effect of other opioids, subsequent encounter |
| T40.3X5A | Adverse effect of methadone, initial encounter |
| T40.3X5D | Adverse effect of methadone, subsequent encounter |
| T40.4X5A | Adverse effect of synthetic narcotics, initial encounter |
| T40.4X5D | Adverse effect of synthetic narcotic, subsequent encounter |
| T40.605A | Adverse effect of unspecified narcotics, initial encounter |
| T40.605D | Adverse effect of unspecified narcotics, subsequent encounter |
| T40.695A | Adverse effect of other narcotics, initial encounter |
| T40.695D | Adverse effect of other narcotics, subsequent encounter |
| **Long-term use of opiates** | |
| Z79.891 | Long-term (current) use of opiate analgesic |
