# Supplementary material for: Improving the management of chronic pain, opioid use, and opioid use disorder in older adults: study protocol for I-COPE study
Source: Trials. 2022 Jul 27;23:602. doi: 10.1186/s13063-022-06537-w (PMC9327217; doi:10.1186/s13063-022-06537-w)

**Additional file 5**

Improving Chicago Older Adult Opioid and Pain Management Through Patient-centered Clinical Decision Support and Project ECHO® (I-COPE) Conversation tool


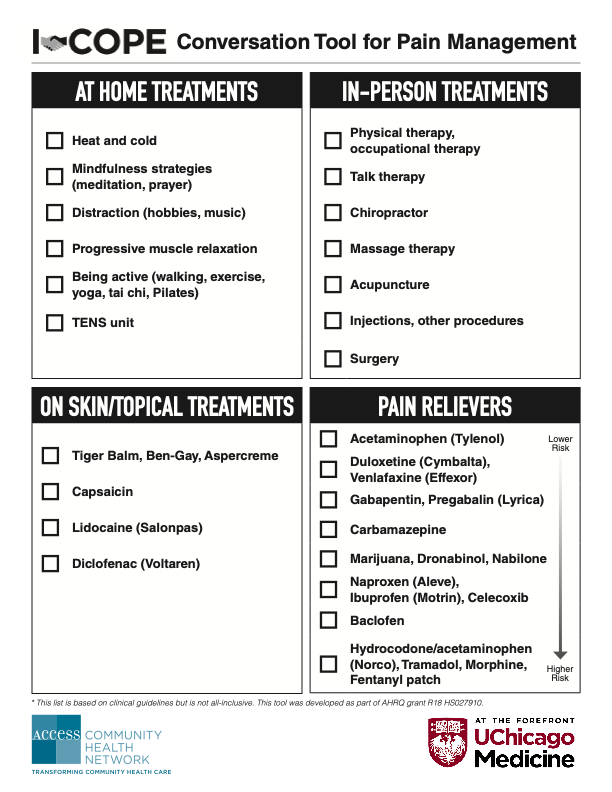

Supplement: Supplementary file 5 — Additional file 5. Improving Chicago Older Adult Opioid and Pain Management Through Patient-centered Clinical Decision Support and Project ECHO (I-COPE) Conversation tool. [file 13063_2022_6537_MOESM5_ESM.docx]
