# Supplementary material for: Improving the management of chronic pain, opioid use, and opioid use disorder in older adults: study protocol for I-COPE study
Source: Trials. 2022 Jul 27;23:602. doi: 10.1186/s13063-022-06537-w (PMC9327217; doi:10.1186/s13063-022-06537-w)
Supplement: Supplementary file 6 — Additional file 6. Notes on sample size calculation and data analysis. [file 13063_2022_6537_MOESM6_ESM.docx]

**Additional file 6**

Notes on sample size calculation and data analysis

**Sample Size.** We calculated the sample size for the effectiveness outcome that we had pre-intervention data for, pain scores using the sample size calculation method for close cohort stepped wedge design by Hooper et al (2016)^25^ and Hooper and Bourke (2015).^26^ We estimated that there are an average of 26 providers and about 600 patients per step. We expect that at least 68% of older adults with pain scores ≥6 experiencing ≥30% pain score reduction due to the intervention. The total sample of 3,040 eligible patients within 5 steps is needed to ensure at least 80% power to detect the difference of 10% at one-sided significance level of 5%. Additional information is available below.

In this pragmatic stepped wedge cluster-randomized study, we are adequately powered to find a difference in the effectiveness outcome of the proportion of older adults with pain scores ≥6 with at least 30% pain reduction within 6 months. Among the total number of clinics (N=37), after we have excluded the two flagship clinics from the stepped wedge design, we have assumed that at least more than 90% will remain in the study. For the remaining 35 clinics, based on electronic health record data, there are about 3,000 older adults with pain scores ≥6, who receive care within a 12-month period. Five steps are needed to implement the I-COPE program to be able to train about 25-27 providers per step. Each step has a size of ~600 eligible patients from 5-9 sites. We expect that at least 68% of older adults with pain scores ≥6 will experience ≥30% pain score reduction due to the intervention, compared to only a 58% in the control arm, based on the results from the SPACE randomized clinical trial.^13^ We calculated the sample size for the stepped-wedge trial adopting the formula^25,26^ by using two design effects due to clustering and repeated assessment respectively. To be conservative, the within-period intracluster correlation (wpICC, i.e., the correlation between assessments of two individuals from the same cluster at the same time) was set to be 0.01 for the binary outcome. The size of wpICC in a cluster trial is usually very small (between 0.01-0.03).^31^ The between-period ICC (bpICC, i.e., the correlation between two individuals from the same cluster at different periods) was set to be 0.06, implying 60% extent of the cluster correlation relative to wpICC, which is suggested to be between 30% and 90%.^32^ The individual autocorrelation coefficient was set to be 0.5, a median of correlation within individual. Due to unequal cluster size, we use the coefficient of variation of 0.41^33^ (median, estimated from a systematic review study) to inflate the clustering design effect.^34^ With the sample size calculation method by Hooper et al (2016)^25^ and Hooper and Bourke (2015)^26^, a total sample of 3,040 with 5 steps was needed to gain at least 80% power to detect the difference of 10% at one-sided significance level of 5%. With a total of >3,400 patients with baseline pain score ≥6 in the 35 sites, the power becomes much bigger.

**Data Analysis.** All analyses will be performed by the University of Chicago Medicine. An intention-to-treat (ITT) analysis principle will be applied to all outcomes. For analyses of the primary effectiveness outcome (proportion of older adults with chronic pain diagnoses and pain scores ≥6 with ≥30% pain reduction), we will use the blended exchangeable correlation structure^27^ via generalized estimating equation (GEE) and generalized linear mixed-effects model (GLMM) to evaluate the intervention effect over 2 years since the beginning of the program implementation. For modeling the intervention effect, because of uncertainty about intervention delivery during the 12-week active implementation phase, we will estimate it using the general delayed treatment effect method^27^. For example, during the transition phase, the intervention is known to be 50% effective, while after the transition phase, the intervention is known to be 100% effective. For modeling the secular trend, we will use three ways: discrete periods, linear trend, and second-order polynomial trend, and choose the best model fit in terms of model fit criteria such as QIC and QICu in GEE, and pseudo-likelihood in GLMM. For modeling the heterogeneity, we will include the cluster random effect, the random cluster-by-time interaction effect, and the random effect for the repeated measures from an individual in a cluster (i.e., site). Following the blended exchangeable correlation structure, we will estimate within-individual ICC, between-period ICC, and within-period ICC. We will adjust for patient-level potential confounders such as age, gender, race/ethnicity and comorbidity scores. In the final model of the whole study period, we will estimate and test the intervention effect at 6 months, which will be considered as a primary analysis at the one-sided significance level of 5%.

For analyses of secondary outcomes, for outcomes that require data collection provided by the intervention, such as reach, adoption, and implementation outcomes, we will provide basic descriptive statistics per clinic per period and over all clinics and all periods. For all other effectiveness outcomes including maintenance outcomes, we will repeat the same analysis methods as for the primary effectiveness outcome. For continuous outcomes such as pain score, we will use the multi-level model, i.e., the linear mixed-effects model (LMM), to assess the intervention effect. Similar to the binary outcome analysis for the expanding cohort stepped-wedge design, we will use the blended exchangeable correlation structure to conduct a LMM and use the same logic as for the binary outcome to model the intervention effect, the secular trend, and the heterogeneity^27^.

To check consistency in the intervention effect, we will conduct subgroup analyses by repeating analyses within each wedge and treat the study as a series of unbalanced parallel cluster trials. For analyses of the two flagship clinics, we will construct a control group within each clinic based on those with chronic pain diagnoses and pain scores ≥ 6 for the 6 months before the intervention. The intervention group will include those after the completion of implementation excluding those in the control group. We will conduct GEE and GLMM for binary outcomes and LMM for continuous outcomes.
